# Gut Microbiota and Metagenomic Advancement in Digestive Disease

**DOI:** 10.1155/2016/4703406

**Published:** 2016-05-10

**Authors:** Jinsheng Yu, Sharon Marsh, Junbo Hu, Wenke Feng, Chaodong Wu

**Affiliations:** ^1^Department of Genetics, Washington University School of Medicine, St. Louis, MO 63110, USA; ^2^Faculty of Pharmacy and Pharmaceutical Sciences, University of Alberta, Edmonton, AB, Canada T6G 2H7; ^3^Department of General Surgery, Tongji Hospital, Huazhong Science & Technology University, Wuhan, Hubei 430030, China; ^4^Department of Medicine, University of Louisville, Louisville, KY 40208, USA; ^5^Department of Nutrition and Food Science, Texas A&M University, Houston, TX 77843, USA

Recent studies have made significant advances in understanding the mechanisms of gut microbiota involved in human health and disease [1, 2]. Now the gut microbiota has been recognized as a key player in a broad spectrum of human diseases from obesity associated liver and cardiovascular diseases to mental development and psychiatric diseases [3, 4]. Accordingly, modulations of gut microbial diversity and composition are expected to improve human health and to provide novel therapeutic modalities for human disease. The gut microbial modulators can be simply specific diets and drinks, natural tea and Chinese herbs, or specialized prebiotics and probiotics.

In this special issue, authors presented a number of very interesting studies on changes of gut microbiota in digestive diseases. These review and original articles of research and clinical studies cover a range of topics, including the pathogenesis of alcoholic and nonalcoholic fatty liver diseases (NAFLD), the outcome of intestinal bacterial translocation in advanced cirrhosis, the gut microbiota changes in an animal colitis model after treatment with a monoclonal antibody, and fecal microbiota transplantation (FMT) in elderly patients with refractory* Clostridium difficile *infection. In these articles, authors have described mechanisms of disease development as well as therapeutic effects of specific antibodies, probiotics, and FMT.

While we know the simple cause of alcoholic fatty liver disease (AFLD), its pathogenesis is complicated and it involves a wide range of changes in host metabolism and gut microbiota. Certainly, removal of the cause (alcohol abuse) is the first treatment for AFLD, and the use of probiotics can ameliorate its development as shown in recent clinical and animal studies [5–7]. Although sharing many pathological and clinical features with AFLD, NAFLD does not have a simple, single cause. Instead, NAFLD is the result of interactions between gut microbiota, host genetics, and diet. There have been a number of studies evaluating the effects and mechanisms of probiotics [8–10], natural tea [11, 12], and Chinese herbs/recipes [13, 14] on AFLD and NAFLD. These studies reported beneficial effects with promising perspectives for future development of novel therapeutic strategies.

However, in light of the nature of gut microbiota being highly diverse and constitutional, built during the early life of individuals, cautious optimism is necessary for any future therapeutic developments aiming at modulations of gut microbiota, as we do not yet know long-term effects from those initial efforts or indeed which of those microbiota and metabolomics changes are contributory, causative, or simply a cofounder in AFLD and NAFLD, or indeed any other condition. Thus far, it is not clear whether the modulations by specific diets and drinks including natural tea, even by probiotics, are able to change the constitutional nature of gut microbiota, although the relative abundance of microbial species is altered upon administrating modulators [12, 13, 15]. Moreover, current analytic strategies on metagenomics data are focused on major changes in gut microbial compositions and have not paid sufficient attention to or have even ignored the changes in minor species with less than 1% abundance. Furthermore, sampling of microbiota in intestinal lumen may not necessarily represent the mucosal portion or the whole population of gut flora [16]. In fact, the true signal and messengers governing the alterations of gut microbiota and host metabolism are largely elusive. Enteric short-chain fatty acids (SCFAs), such as acetate, propionate, and butyrate, are fermentation metabolites by intestinal bacteria. Recent studies have suggested a messenger role of SCFAs in several human diseases including metabolic syndrome [17] and autism [4]. In short, careful designs of future studies should take these factors and host genetic background under consideration to discover the real effectors and to discern the true effects of gut microbial modulators in alcoholic and nonalcoholic fatty liver disease and other human diseases.

*Jinsheng Yu* *Jinsheng Yu* *Sharon Marsh* *Sharon Marsh* *Junbo Hu* *Junbo Hu* *Wenke Feng* *Wenke Feng* *Chaodong Wu* *Chaodong Wu*

## References

[1] Faith J. J., Guruge J. L., Charbonneau M. (2013). The long-term stability of the human gut microbiota. *Science*.

[2] Kau A. L., Ahern P. P., Griffin N. W., Goodman A. L., Gordon J. I. (2011). Human nutrition, the gut microbiome and the immune system. *Nature*.

[3] Goyal M. S., Venkatesh S., Milbrandt J., Gordon J. I., Raichle M. E. (2015). Feeding the brain and nurturing the mind: linking nutrition and the gut microbiota to brain development. *Proceedings of the National Academy of Sciences*.

[4] MacFabe D. F. (2015). Enteric short-chain fatty acids: microbial messengers of metabolism, mitochondria, and mind: implications in autism spectrum disorders. *Microbial Ecology in Health and Disease*.

[5] Dhiman R. K., Rana B., Agrawal S. (2014). Probiotic VSL#3 reduces liver disease severity and hospitalization in patients with cirrhosis: a randomized, controlled trial. *Gastroenterology*.

[6] Kirpich I. A., Solovieva N. V., Leikhter S. N. (2008). Probiotics restore bowel flora and improve liver enzymes in human alcohol-induced liver injury: a pilot study. *Alcohol*.

[7] Bull-Otterson L., Feng W., Kirpich I. (2013). Metagenomic analyses of alcohol induced pathogenic alterations in the intestinal microbiome and the effect of *Lactobacillus rhamnosus* GG treatment. *PLoS ONE*.

[8] Alisi A., Bedogni G., Baviera G. (2014). Randomised clinical trial: the beneficial effects of VSL#3 in obese children with non-alcoholic steatohepatitis. *Alimentary Pharmacology and Therapeutics*.

[9] Ritze Y., Bárdos G., Claus A. (2014). *Lactobacillus rhamnosus* GG protects against non-alcoholic fatty liver disease in mice. *PLoS ONE*.

[10] Xu R.-Y., Wan Y.-P., Fang Q.-Y., Lu W., Cai W. (2012). Supplementation with probiotics modifies gut flora and attenuates liver fat accumulation in rat nonalcoholic fatty liver disease model. *Journal of Clinical Biochemistry and Nutrition*.

[11] Santamarina A. B., Oliveira J. L., Silva F. P. (2015). Green tea extract rich in epigallocatechin-3-gallate prevents fatty liver by AMPK activation via LKB1 in mice fed a high-fat diet. *PLoS ONE*.

[12] Axling U., Olsson C., Xu J. (2012). Green tea powder and *Lactobacillus plantarum* affect gut microbiota, lipid metabolism and inflammation in high-fat fed C57BL/6J mice. *Nutrition and Metabolism*.

[13] Lin P., Lu J., Wang Y. (2015). Naturally occurring stilbenoid TSG reverses non-alcoholic fatty liver diseases via gut-liver axis. *PLoS ONE*.

[14] Cheng Y., Wang H.-H., Hu Y.-Y. (2011). Effect of jianpi huoxue recipe on gut flora in rats with alcoholic fatty liver induced by Lieber-DeCarli liquid diet. *Zhongguo Zhong Xi Yi Jie He Za Zhi*.

[15] Baldwin J., Collins B., Wolf P. G. (2016). Table grape consumption reduces adiposity and markers of hepatic lipogenesis and alters gut microbiota in butter fat-fed mice. *The Journal of Nutritional Biochemistry*.

[16] Yasuda K., Oh K., Ren B. (2015). Biogeography of the intestinal mucosal and lumenal microbiome in the rhesus macaque. *Cell Host and Microbe*.

[17] den Besten G., Bleeker A., Gerding A. (2015). Short-chain fatty acids protect against high-fat diet-induced obesity via a PPARgamma-dependent switch from lipogenesis to fat oxidation. *Diabetes*.

